# Whole-body net protein balance plateaus in response to increasing protein intakes during post-exercise recovery in adults and adolescents

**DOI:** 10.1186/s12986-018-0301-z

**Published:** 2018-09-24

**Authors:** Michael Mazzulla, Kimberly A. Volterman, Jeff E. Packer, Denise J. Wooding, Jahmal C. Brooks, Hiroyuki Kato, Daniel R. Moore

**Affiliations:** 1 Faculty of Kinesiology and Physical Education, University of Toronto, Toronto, ON Canada; 2 Frontier Research Laboratories, Institute for Innovation, Ajinomoto Co., Inc, Kawasaki, Japan

**Keywords:** Amino acids, Athlete, Growth, Lean body mass, Maximal anabolic response, Muscle protein synthesis, Postprandial, Youth

## Abstract

**Background:**

Muscle protein synthesis and muscle net balance plateau after moderate protein ingestion in adults. However, it has been suggested that there is no practical limit to the anabolic response of whole-body net balance to dietary protein. Moreover, limited research has addressed the anabolic response to dietary protein in adolescents. The present study determined whether whole-body net balance plateaued in response to increasing protein intakes during post-exercise recovery and whether there were age- and/or sex-related dimorphisms in the anabolic response.

**Methods:**

Thirteen adults [7 males (M), 6 females (F)] and 14 adolescents [7 males (AM), 7 females (AF) within ~ 0.4 y from peak height velocity] performed ~ 1 h variable intensity exercise (i.e., Loughborough Intermittent Shuttle Test) prior to ingesting hourly mixed meals that provided a variable amount of protein (0.02–0.25 g·kg^− 1^·h^− 1^) as crystalline amino acids modeled after egg protein. Steady-state protein kinetics were modeled noninvasively with oral L-[1-^13^C]phenylalanine. Breath and urine samples were taken at plateau to determine phenylalanine oxidation and flux (estimate of protein breakdown), respectively. Whole-body net balance was determined by the difference between protein synthesis (flux – oxidation) and protein breakdown. Total amino acid oxidation was estimated from the ratio of urinary urea/creatinine.

**Results:**

Mixed model biphasic linear regression explained a greater proportion of net balance variance than linear regression (all, *r*^2^ ≥ 0.56; *P* < 0.01), indicating an anabolic plateau. Net balance was maximized at ~ 0.15, 0.12, 0.12, and 0.11 g protein·kg^− 1^·h^− 1^ in M, F, AM, and AF, respectively. When collapsed across age, the y-intercept (net balance at very low protein intake) was greater (overlapping CI did not contain zero) in adolescents vs. adults. Urea/creatinine excretion increased linearly (all, *r* ≥ 0.76; *P* < 0.01) across the range of protein intakes. At plateau, net balance was greater (*P* < 0.05) in AM vs. M.

**Conclusions:**

Our data suggest there is a practical limit to the anabolic response to protein ingestion within a mixed meal and that higher intakes lead to deamination and oxidation of excess amino acids. Consistent with a need to support lean mass growth, adolescents appear to have greater anabolic sensitivity and a greater capacity to assimilate dietary amino acids than adults.

## Background

Dietary protein ingestion facilitates post-exercise recovery [1] and supports the growth and maintenance of lean body mass throughout the lifespan [2]. It is well established that protein intake enhances muscle protein net balance (for reviews see: [3, 4]) primarily through the stimulation of muscle protein synthesis [5], which plateaus after ingestion of ~ 20 g protein [6, 7] or ~ 0.25–0.30 g·kg^− 1^ in healthy adults [8]. When muscle protein synthesis exceeds muscle protein breakdown, the resulting positive net balance facilitates lean mass accretion. However, the capacity to assimilate dietary amino acids into new proteins is greater when considering all body protein pools such as the labile pool within the splanchnic bed [9]. This has led to the suggestion that there is no practical limit to the anabolic response to protein ingestion such that increasing protein intake at each meal is the most efficient way to maximize whole-body net balance over a 24-h time period [10–12]. Indeed, the reported habitual protein intakes of some active populations far exceed the recommended daily allowance of 0.8 g·kg^− 1^·d^− 1^ [13] and even general sports nutrition consensus statements (i.e., 1.2–2.0 g·kg^− 1^·d^− 1^) [14]. However, dietary protein consumed in excess of the rate at which it can be incorporated into body protein stimulates irreversible amino acid oxidation [7] and a subsequent increase in urinary urea production [15]. Therefore, to optimize dietary protein efficiency during post-exercise recovery, it is important to establish a protein intake that maximizes net balance while simultaneously minimizing oxidation.

Post-exercise protein ingestion increases whole-body leucine and protein balance in a dose-dependent manner in children, although evidence of any apparent plateau with moderate protein ingestion (i.e., 0–0.35 g·kg^− 1^) was not observed [16, 17]. Notwithstanding the consistent linear growth of childhood, adolescence marks a period of rapid lean mass growth that is only superseded in rate, but not in magnitude, by that experience within the first year of life [18]. Moreover, lean mass accrual during this critical growth period is further influenced by physical activity, as active adolescents have a greater lean body mass than their sedentary counterparts [19]. The accelerated lean mass growth of adolescence compared to childhood may be supported by a greater efficiency of dietary protein utilization [20] which could suggest that, at least in non-exercising youth, the adolescent growth spurt may sensitize the body to low doses of amino acids and/or enhance the capacity for net protein retention at higher doses. Despite the interaction between protein ingestion and exercise to support anabolism in adults [3, 4], no studies, to our knowledge, have addressed the impact that age (e.g., adolescence vs. adulthood) may have on maximizing whole-body net balance during post-exercise recovery. Thus, it is unclear whether adolescence enhances the sensitivity toward and/or capacity to assimilate dietary protein to enhance whole-body net balance after exercise when compared to weight-stable adults.

Traditionally, protein metabolism is studied using primed constant infusions of stable isotope amino acid tracers with concomitant measurements of isotopic steady state in plasma [21]. The invasiveness of these techniques, however, limits their practical use in vulnerable groups (e.g., adolescents). Accordingly, oral tracer models such as the indicator amino acid oxidation (IAAO) technique, which has been used to determine protein requirements in humans [22], have expanded the noninvasive study of protein metabolism since they have been shown to produce the required isotopic steady state in urine similar to steady states measured in plasma [23], the latter of which is generally used as a surrogate precursor pool enrichment in standard whole-body tracer methods [21]. Although knowledge of the true precursor pool for protein synthesis (i.e., hepatic phenylalanyl-tRNA enrichment) would be required to clearly determine the partitioning of the indicator amino acid (i.e., phenylalanine) between synthesis and oxidation, the IAAO applies mixed modeling biphasic analysis of graded test protein intakes that is uninfluenced by any slight systematic over/under-estimation of protein kinetics that may arise from surrogate precursor pool enrichments, such as urine, and thus can generate robust breakpoints corresponding to the estimated average requirement of a population [24]. Therefore, the present study utilized the minimally invasive IAAO technique to determine whether whole-body net balance plateaued in response to increasing protein intakes during post-exercise recovery in active adults and adolescents, and whether there was age- and/or sex-related dimorphisms in the anabolic response. We hypothesized that: i) whole-body net balance would increase up to a plateau during post-exercise recovery in both populations, and; ii) due to a presumably greater sensitivity to dietary protein during pubertal growth, adolescents would reach a plateau in net balance at a lower protein intake and/or exhibit a greater net balance at plateau when compared to adults.

## Methods

### Participants

Thirteen adults [7 males (M), 6 females (F)] and 14 adolescents [7 males (AM), 7 females (AF) between − 0.5 to + 1.0 y from peak height velocity] were included in the present study (Table 1). Peak height velocity is the period during adolescence in which maximal statural growth occurs and robust physiological changes are observed, and by using previously published sex-specific multiple-regression equations [25] it is possible to assess a child’s biological maturity by calculating at what age their peak height velocity is predicted to occur. The data presented herein are part of a secondary analysis to the primary outcome in previously published studies [26, 27]. Prior to enrolment, the Physical Activity Readiness Questionnaire was used to assess participant health risks. Training status was characterized using the International Physical Activity Questionnaire and participant VO_2max_ was predicted using the Leger Multistage Fitness Test [28]. All participants were required to be healthy and have habitual activity levels ≥45 min·d^− 1^ on 5 d·wk.^− 1^.

**Table 1:** Participant characteristics

	M (*n* = 7)^a^	F (*n* = 6)^a^	AM (*n* = 7)	AF (*n* = 7)
Age (y)	23 ± 1^a^	21 ± 1^a^	14 ± 1^b^	12 ± 1^b^
Weight (kg)	82 ± 6^a^	69 ± 4^b^	57 ± 6^c^	52 ± 10^c^
FFM (kg)	71 ± 6^a^	54 ± 3^b^	49 ± 7^b,c^	42 ± 9^c^
VO_2max_ (ml O_2_·kg^− 1^·min^− 1^)	52 ± 6^*^	47 ± 1	54 ± 2^*^	48 ± 2
Daily EE (kcal·kg^− 1^·d^− 1^)	52 ± 4^a^	39 ± 2^b^	50 ± 7^a^	40 ± 6^b^

Participant data are mean ± SD and were analyzed using a two-way (age × sex) ANOVA

Different superscript letters denote significant between-group differences (all, *P* < 0.05); * denotes main effect of sex (*P* < 0.01)

^a^Previously published subject data [26, 27]

*AF* adolescent females, *AM* adolescent males, *EE* energy expenditure, *F* adult females, *FFM* fat-free mass, *M* adult males, *VO*_*2max*_ maximal oxygen uptake

### Experimental protocol

The present study design was based on the IAAO technique and has been described in detail elsewhere [26, 27]. Briefly, daily energy expenditure was estimated over 3 d by armband accelerometry (SenseWear, BodyMedia, Pittsburgh, PA). Fat-free mass (FFM) was determined by air displacement plethysmography (BodPod®, Cosmed USA, Chicago, IL) after avoiding food, water, and exercise for ≥4 h. For 2 d prior to each metabolic trial (see below), participants were provided with an adaptation diet consisting of commercially available, prepackaged foods that provided 1.2 g protein·kg^− 1^·d^− 1^ and sufficient energy to match their habitual expenditure as measured by 3-d accelerometer record.

### Metabolic trial

**Fig. 1:**
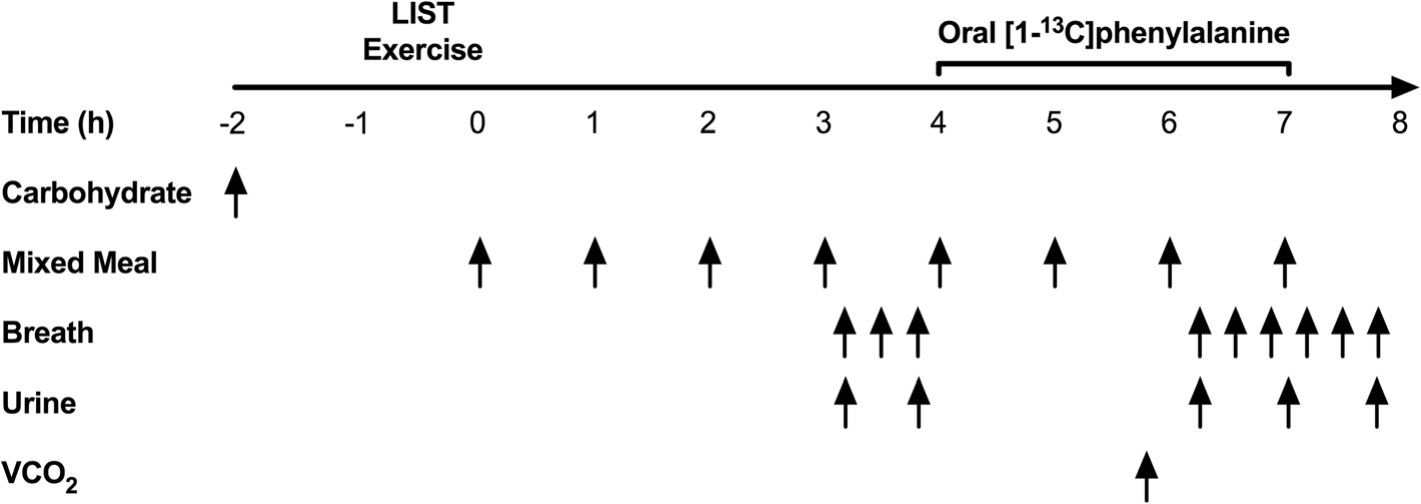
Metabolic trial day schematic. Following an overnight fast, participants completed the LIST and subsequently consumed eight hourly isoenergetic mixed meals with protein provided as crystalline amino acids. The final four test drinks contained L-[1-^13^C]phenylalanine to model steady-state phenylalanine kinetics. Breath and urine samples were collected at baseline and isotopic plateau and were analyzed for ^13^CO_2_ enrichment and L-[1-^13^C]phenylalanine enrichment, respectively. Steady-state CO_2_ production (VCO_2_) was measured for 20 min after the fifth test drink by indirect calorimetry. Urinary urea and creatinine excretion was determined by enzymatic assay

Each participant performed 5–8 metabolic trials consisting of two components: a modified version of the Loughborough Intermittent Shuttle Test (LIST) and a subsequent 8-h postprandial period with a variable protein intake (see Fig. 1 for trial overview). Each participant was randomized to consume unique protein intakes within pre-defined ranges and were requested to be available to complete ≥4 metabolic trials, as this is the minimum number of trials required to achieve robust breakpoints in the primary outcome (i.e., [^13^C] tracer excretion). With a target of *n* = 42 unique intakes, adults were randomly assigned to consume a protein intake from seven pre-defined ranges (i.e., 0.017–0.038 g·kg^− 1^·h^− 1^; 0.042–0.063 g·kg^− 1^·h^− 1^; 0.067–0.088 g·kg^− 1^·h^− 1^; 0.092–0.113 g·kg^− 1^·h^− 1^; 0.117–0.138 g·kg^− 1^·h^− 1^; 0.142–0.163 g·kg^− 1^·h^− 1^; 0.167–0.188 g·kg^− 1^·h^− 1^). However, due to time constraints and scheduling conflicts participants completed a minimum of five and a maximum of eight trials. In addition, based on an interim analysis, some participants were additionally randomized to intakes 0.188 g/kg/h to better define the breakpoint. With a target of *n* = 42 unique intakes, adolescents were randomly assigned to consume a protein intake from six pre-defined ranges (i.e., 0.0167–0.046 g·kg^− 1^·h^− 1^; 0.052–0.081 g·kg^− 1^·h^− 1^; 0.081–0.114 g·kg^− 1^·h^− 1^; 0.122–0.151 g·kg^− 1^·h^− 1^; 0.158–0.186 g·kg^− 1^·h^− 1^, 0.193–0.222 g·kg^− 1^·h^− 1^) with all participants completing six trials. Adult female participants completed trials during the predicted luteal phase, which was defined as the second half of the menstrual cycle. Following an overnight fast, participants consumed a protein-free liquid carbohydrate beverage (1 g·kg^− 1^) as a 1:1 ratio of maltodextrin (Polycal™, Nutricia, Amsterdam, Netherlands) and sports drink powder (Gatorade Endurance Formula, PepsiCo, Purchase, NY) before reporting to the laboratory. The purpose of the beverage was to help replenish overnight fasted losses of liver glycogen and to provide exogenous carbohydrate to fuel the exercise stimulus. Approximately 1 h after carbohydrate ingestion, participants completed the LIST according to a previously described exercise protocol [26, 27]. In an effort to standardize the relative metabolic work required during the LIST, the distance of the course for each group was adjusted according to the expected average height (i.e., M = 18.5 m; F = 17 m; AM/AF = 16 m).

Immediately following the LIST, participants consumed eight hourly isoenergetic mixed meals providing a variable amount of protein (0.02–0.25 g·kg^− 1^·h^− 1^) and fat (0.08–0.18 g·kg^− 1^·h^− 1^) with 0.42 g·kg^− 1^·h^− 1^ of carbohydrate per meal. Protein was provided as crystalline amino acids (Ajinomoto North America, Raleigh, NC) modeled after the composition of egg protein [29]. The fifth meal contained priming doses of 0.176 mg·kg^− 1^ Na^13^CO_2_ and 1.86 mg·kg^− 1^ L-[1-^13^C]phenylalanine (Cambridge Isotope Laboratories, Tewksbury, MA) [24]. Subsequent hourly meals (i.e., meals 6–8) contained 0.46 mg·kg^− 1^·h^− 1^ L-[1-^13^C]phenylalanine to maintain isotopic steady state and a constant oral infusion of phenylalanine. Excess tyrosine (3.33 mg·kg^− 1^·h^− 1^) was provided to ensure metabolic partitioning of the phenylalanine carboxyl group into either synthesis or oxidation [30]. The primed oral infusion of L-[1-^13^C]phenylalanine has been previously shown to produce the isotopic steady-state condition required for amino acid flux and oxidation determination within an 8-h postprandial period [23] and was used herein to establish a breakpoint in whole-body net balance (see below).

### Breath and urine samples

Breath and urine samples were collected at baseline and isotopic plateau and were analyzed for ^13^CO_2_ enrichment by continuous-flow isotope ratio mass spectrometry and L-[1-^13^C]phenylalanine enrichment by liquid chromatography-tandem mass spectrometry, respectively, as previously described [29]. Steady-state CO_2_ production (VCO_2_) was measured for 20 min after the fifth test drink by indirect calorimetry as previously described [29]. Urinary urea and creatinine excretion was determined by enzymatic assay according to manufacturer’s instructions (BioAssay Systems, QuantiChrom DIUR-100, DICT-500, Hayward, CA). Amino acid deamination and oxidization is reflected by an increase in urinary urea production [15] and urinary creatinine excretion is related to FFM [31]. Thus, the ratio of urea/creatinine was used herein as an estimate of total amino acid oxidation.

### Tracer kinetics

Oral amino acid tracers have been used to model whole-body protein metabolism noninvasively in adults [23] and adolescents [20]. Bross et al. [23] demonstrated that a primed oral dose of L-[1-^13^C]phenylalanine produced isotopic steady states in urine, plasma, and breath CO_2_ similar to steady states produced by intravenous phenylalanine tracer models [32, 33]. Furthermore, administration of an oral L-[1-^13^C]phenylalanine tracer yields similar enrichments of [^13^C] phenylalanine in urine as compared to plasma, which subsequently obviates the necessity of concomitant blood sampling [23]. Thus, the stable isotope L-[1-^13^C]phenylalanine is an acceptable oral tracer in studies of amino acid flux and oxidation using urinary (rather than plasma) tracer enrichment, which makes it ideally suited for use in vulnerable populations such as children and adolescents.

The isotopic enrichment of L-[1-^13^C]phenylalanine in urine (as an estimate of plasma enrichment) [23] and ^13^CO_2_ breath enrichment was calculated using standard equations developed by Matthews et al. [34] and Rosenblatt et al. [35]. Phenylalanine flux (PheRa; μmol·kg^− 1^·h^− 1^) was measured from L-[1-^13^C]phenylalanine tracer dilution in the urinary pool at isotopic steady state and calculated as follows:

$$ \mathrm{PheRa}=\mathrm{i}\cdotp \left(\frac{{\mathrm{E}}_{\mathrm{i}}}{{\mathrm{E}}_{\mathrm{u}}}\right)-I $$

Where i is the rate of L-[1-^13^C] phenylalanine ingested (μmol·kg^− 1^·h^− 1^); I is the rate of L-phenylalanine ingested (μmol·kg^− 1^·h^− 1^); E_i_ and E_u_ are the isotopic enrichments as mole fractions of the test drink and urinary phenylalanine, respectively, at isotopic plateau.

The rate of ^13^CO_2_ excretion in exhaled breath (F^13^CO_2_; μmol·kg^− 1^·h^− 1^) was calculated as follows:

$$ {\mathrm{F}}^{13}{\mathrm{CO}}_2=\left({\mathrm{V}}_{{\mathrm{CO}}_2}\right)\cdotp \left({\mathrm{E}}_{{\mathrm{CO}}_2}\right)\cdotp (44.6)\cdotp (60)\cdotp {\mathrm{BW}}^{-1}\cdotp (0.82)\cdotp (100) $$

Where VCO_2_ is the CO_2_ production rate (mL·min^−1^); E_CO2_ is the ^13^CO_2_ enrichment in expired breath at isotopic steady state (atom percent excess); BW is the body weight (kg) of the participants. The constants 44.6 μmol·mL^− 1^ and 60 min·h^− 1^ were used to convert F_CO2_ to μmol·h^− 1^. The factor 0.82 is the correction factor for CO_2_ retained in the bicarbonate pool of the body in the fed state [34].

The rate of phenylalanine oxidation (PheOx; μmol·kg^− 1^·h^−1^) was calculated as a ratio of ^13^CO_2_ excretion in exhaled breath and urinary L-[1-^13^C]phenylalanine enrichment as follows:

$$ \mathrm{PheOx}={\mathrm{F}}^{13}{\mathrm{CO}}_2\cdotp {\left(\frac{1}{\mathrm{Eu}}-\frac{1}{\mathrm{Ei}}\right)}^{-1}\times 100 $$

Protein breakdown was estimated by the rate of appearance of phenylalanine in the urine and protein synthesis was calculated as the difference between phenylalanine flux and oxidation (see above) [34]. Whole-body protein net balance (converted to mg·kg^−1^·h^−1^) was calculated as the difference between protein synthesis and protein breakdown.

### Statistical analysis

To determine whether the relationship between protein intake and whole-body net balance was better explained by linear or biphasic regression, the *r*^2^ of the linear regression mixed model (described below) was compared to the *r*^2^ of the biphasic linear regression mixed model (described below), with the highest *r*^2^ identifying the preferred model. The linear mixed model used participants as a random variable using PROC MIXED (SAS University Edition, Version 9.4, Toronto, Canada) to determine the *r*^2^. If the data conformed to a biphasic model, breakpoint analysis of the net balance data using a biphasic linear regression mixed model (SAS University Edition, Version 9.4, Toronto, Canada) was performed in agreement with previous studies [24, 36, 37] to determine estimates of the mean protein intake required to establish a plateau in net balance. The upper 95% CI for the breakpoint was calculated using Fieller’s Theorem as previously described [36]. Data for the breakpoint analyses are presented as mean ± 95% CI. To establish whether the breakpoint differed between groups, we determined the extent of overlapping CI for the breakpoints by the following equation:

$$ \left({\mathrm{Breakpoint}}_1-{\mathrm{Breakpoint}}_2\right)\pm 1.96\ \left[\left[\mathrm{square}\ \mathrm{root}\right]\left({{\mathrm{SE}}_1}^2+{{\mathrm{SE}}_2}^2\right)\right] $$

whereby the null hypothesis was rejected if the interval did not contain zero [27]. To establish whole-body net balance at a very low protein intake, we determined the extent of overlapping CI for the mixed model biphasic y-intercept by the following equation:

$$ \left(\mathrm{y}\hbox{-} {\mathrm{intercept}}_1-\mathrm{y}\hbox{-} {\mathrm{intercept}}_2\right)\pm 1.96\ \left[\left[\mathrm{square}\ \mathrm{root}\right]\left({{\mathrm{SE}}_1}^2+{{\mathrm{SE}}_2}^2\right)\right] $$

whereby the null hypothesis was rejected if the interval did not contain zero. Linear correlation (IBM SPSS Statistics, Version 24, Armonk, NY) was used to identify a relationship between urea/creatinine excretion and protein intake. The whole-body net balance breakpoint was identified for each analysis and a two-way ANOVA (IBM SPSS Statistics, Version 24, Armonk, NY) was used to identify main effects of age, sex, and age × sex interactions for the mean net balance values above the breakpoint to assess between-group differences. Where significant interactions were identified, a Bonferroni corrected t-test (IBM SPSS Statistics, Version 24, Armonk, NY) was used to compare net balance means above the breakpoint. Significance was set at *P* ≤ 0.05.

## Results

### Whole-body net balance biphasic linear regression

**Fig. 2:**
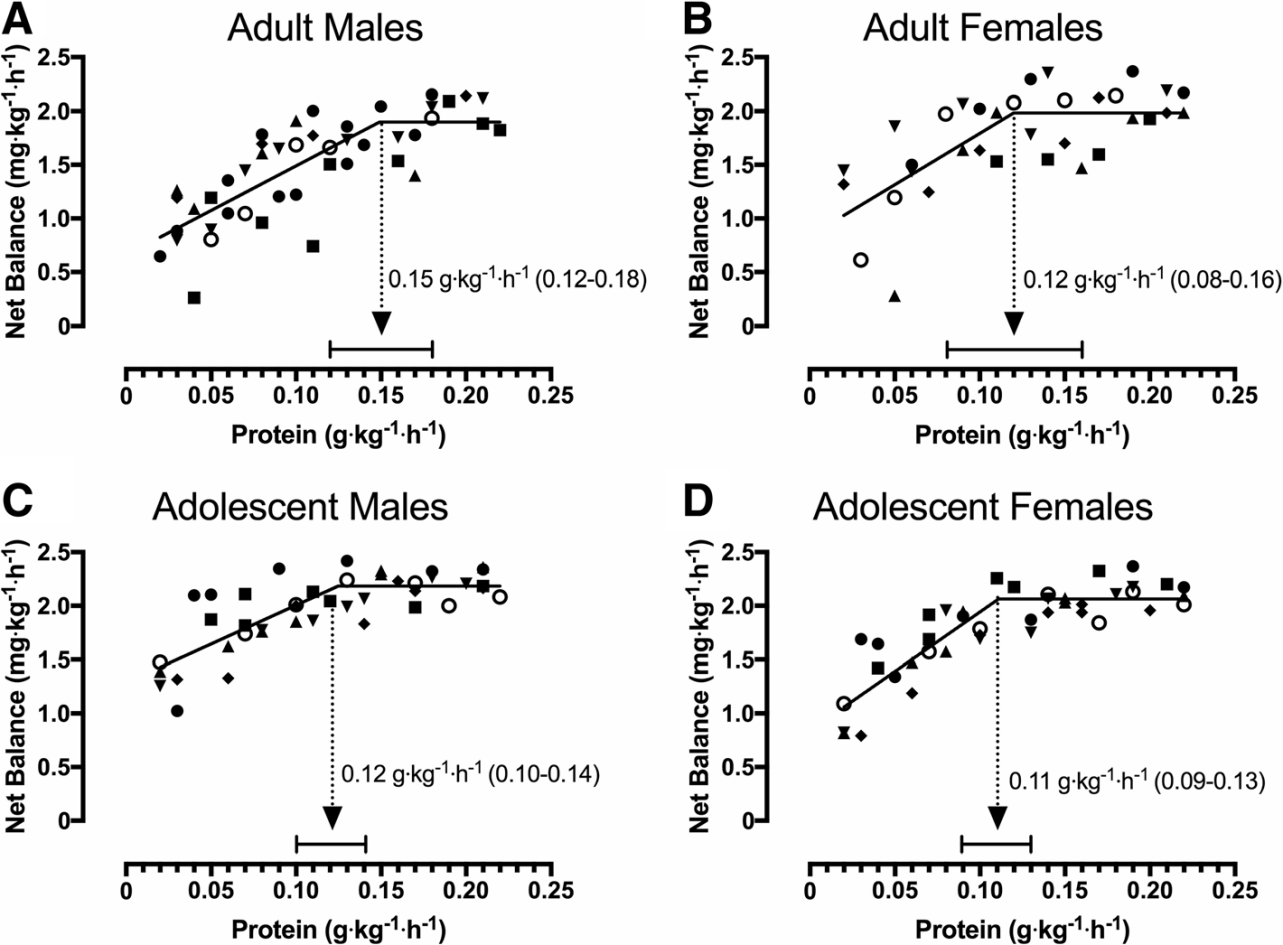
Whole-body net balance plateau in response to increasing protein intakes following a bout of variable intensity exercise. **a**: Adult Males (*n* = 7; total = 45 trials); **b**: Adult Females (*n* = 6; total = 36 trials); **c**: Adolescent Males (*n* = 7; total = 42 trials); **d**: Adolescent Females (*n* = 7; total = 42 trials). Each participant completed 5–8 metabolic trials and individual values for each participant are represented by different symbols. Data are mean ± 95% CI

Mixed model biphasic linear regression explained a greater proportion of net balance variance than linear regression (Table 2), indicating an anabolic plateau. Whole-body net balance increased up to a plateau in all groups (Fig. 2) with no differences (all comparisons, overlapping CI contained zero) in the breakpoint protein intake. When collapsed across age, the breakpoint protein intake was not different between adults (0.13 ± 0.03 g·kg^− 1^·h^− 1^) and adolescents (0.12 ± 0.01 g·kg^− 1^·h^− 1^). The y-intercept (i.e., net balance at very low protein intake) was greater (all comparisons, overlapping CI did not contain zero) in AM (1.2 ± 0.06 mg·kg^− 1^·h^− 1^) when compared to M (0.46 ± 0.09 mg·kg^− 1^·h^− 1^), F (0.35 ± 0.07 mg·kg^− 1^·h^− 1^), and AF (0.67 ± 0.05 mg·kg^− 1^·h^− 1^). When collapsed across age, adolescents (0.87 ± 0.04 mg·kg^− 1^·h^− 1^) had a greater (overlapping CI did not contain zero) y-intercept vs. adults (0.44 ± 0.07 mg·kg^− 1^·h^− 1^).

**Table 2:** *r*^2^ comparison between linear and biphasic regression^a^

	Group	Linear *r*^2^	Biphasic *r*^2^
Protein intake	M	0.56	0.68
(g·kg^−1^·h^−1^)	F	0.43	0.56
	AM	0.54	0.67
	AF	0.64	0.80
Protein intake	M	0.56	0.69
(g·kgFFM^−1^·h^−1^)	F	0.45	0.56
	AM	0.43	0.55
	AF	0.66	0.82

^a^*r*^2^ values for mixed model linear regression and mixed model biphasic linear regression were compared to determine the preferred model for analysis (higher *r*^2^ = preferred model)

*AF* adolescent females, *AM* adolescent males, *F* females, *M* males

When normalized to FFM, whole-body net balance increased up to a plateau in all groups at a protein intake corresponding to 0.17 ± 0.05, 0.14 ± 0.04, 0.14 ± 0.04, and 0.12 ± 0.02 g·kgFFM^− 1^·h^− 1^ in M, F, AM, and AF, respectively, with no differences (all comparisons, overlapping CI contained zero) in the breakpoint intake. When collapsed across age, the breakpoint protein intake was not different (overlapping CI contained zero) between adults (0.16 ± 0.03 g·kgFFM^− 1^·h^− 1^) and adolescents (0.13 ± 0.02 g·kgFFM^− 1^·h^− 1^). The y-intercept was greater (all comparisons, overlapping CI did not contain zero) in AM (1.3 ± 0.08 mg·kgFFM^− 1^·h^− 1^) when compared to M (0.54 ± 0.1 mg·kgFFM^− 1^·h^− 1^), F (0.33 ± 0.06 mg·kgFFM^− 1^·h^− 1^), and AF (0.77 ± 0.04 mg·kgFFM^− 1^·h^− 1^). When collapsed across age, adolescents (1.1 ± 0.05 mg·kgFFM^− 1^·h^− 1^) had greater (overlapping CI did not contain zero) y-intercept vs. adults (0.45 ± 0.08 mg·kgFFM^− 1^·h^− 1^).

### Urinary urea/creatinine excretion

**Fig. 3:**
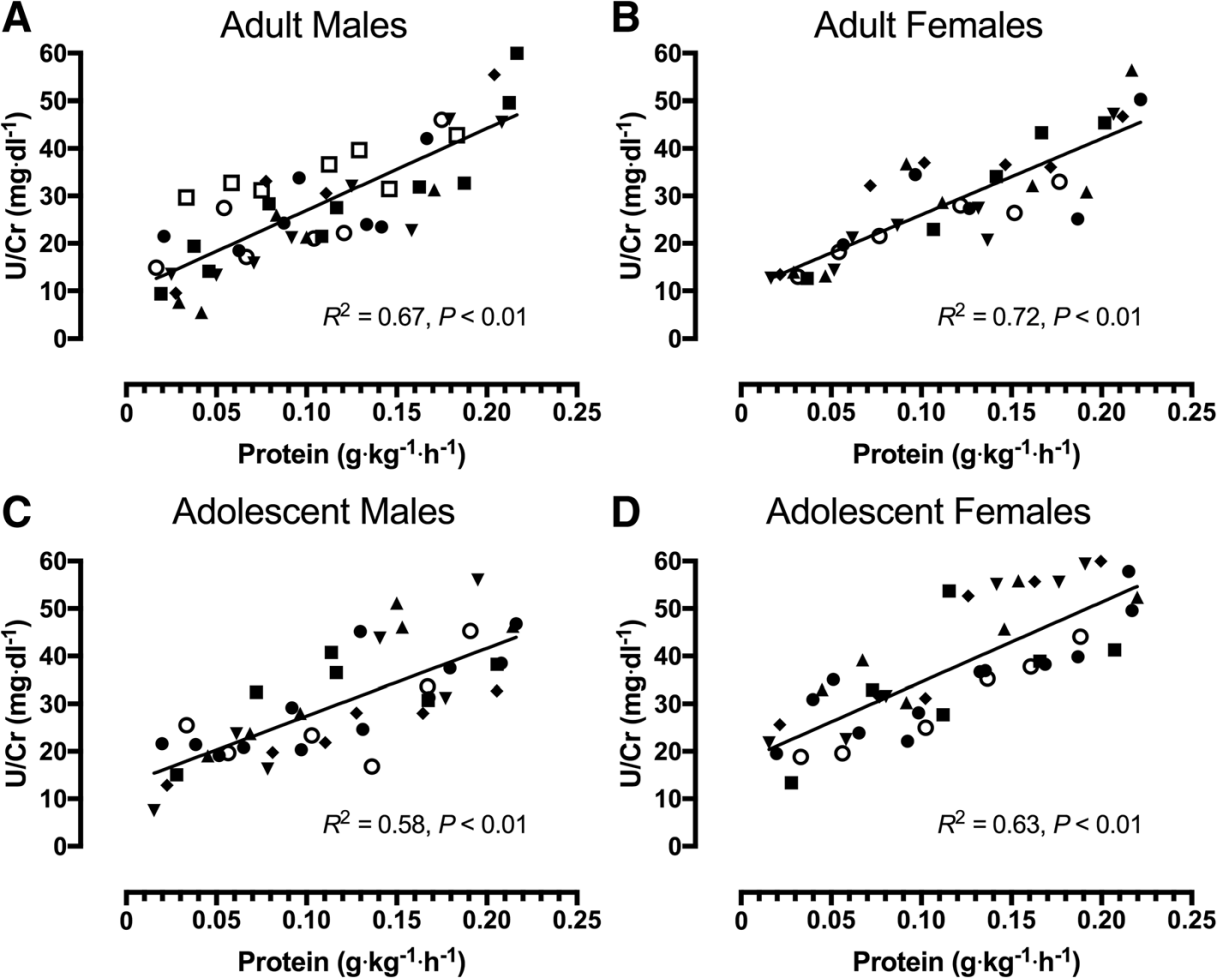
Urea/creatinine excretion in response to increasing protein intakes following a bout of variable intensity exercise. **a**: Adult Males (*n* = 7; total = 45 trials); **b**: Adult Females (*n* = 6; total = 36 trials); **c**: Adolescent Males (*n* = 7; total = 42 trials); **d**: Adolescent Females (*n* = 7; total = 42 trials). Each participant completed 5–8 metabolic trials and individual values for each participant are represented by different symbols

Urinary urea/creatinine excretion (as an estimate of total amino acid oxidation) increased linearly (all, *r* ≥ 0.76; *P* < 0.01) with protein intake in all groups (Fig. 3).

### Whole-body net balance at plateau

**Fig. 4:**
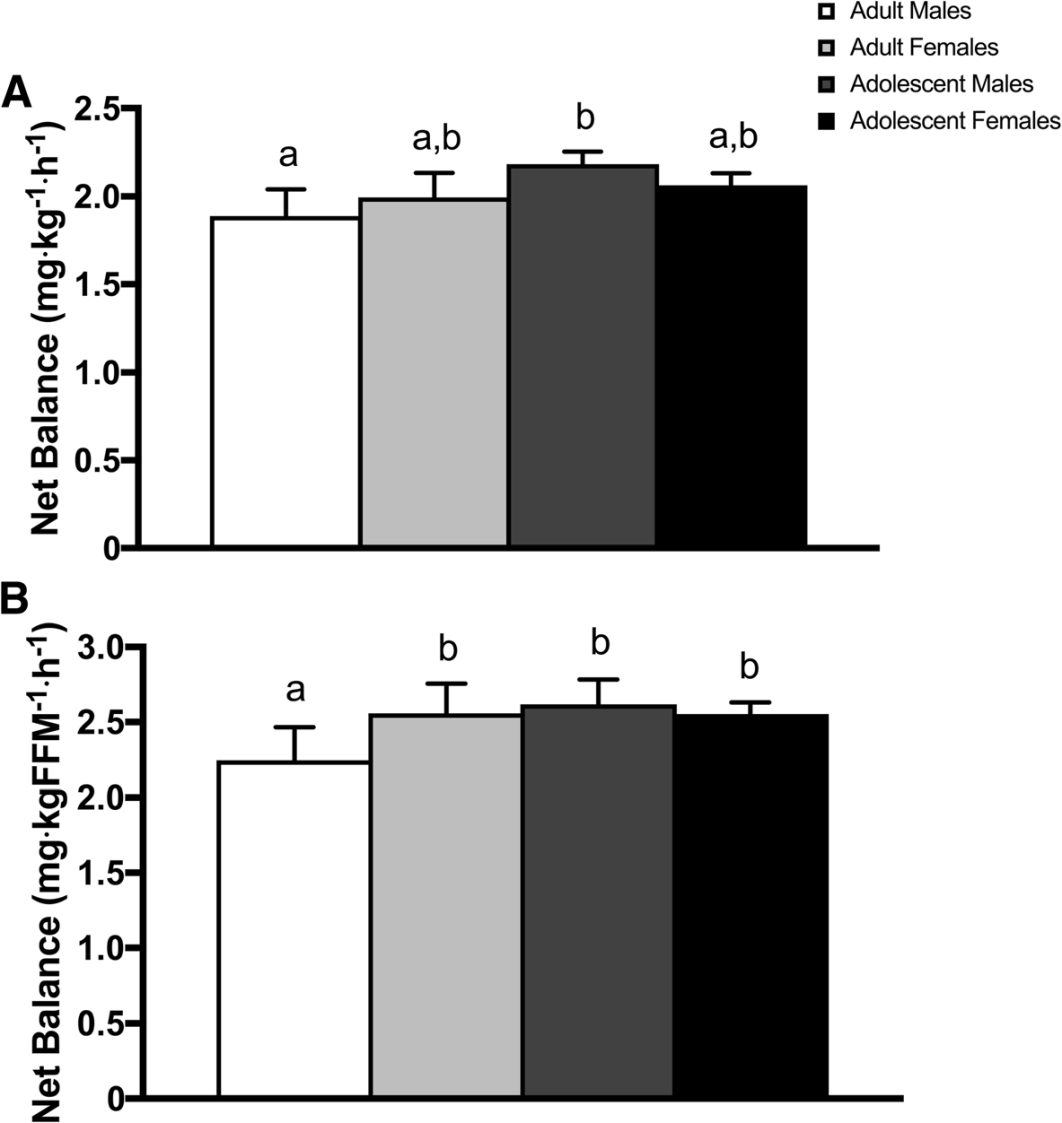
Whole-body net balance at plateau following a bout of variable intensity exercise when (**a**) normalized to body mass and (**b**) normalized to fat-free mass. Data are mean ± 95% CI. Different letters denote significant between-group differences (all, *P* < 0.05)

There was a main effect of age (*P* < 0.01) and an age × sex interaction (*P* = 0.04) for whole-body net balance at plateau (Fig. 4). AM (~ 2.2 mg·kg^− 1^·h^− 1^) had a greater (*P* < 0.05) net balance at plateau than M (~ 1.9 mg·kg^− 1^·h^− 1^) with no further differences (*P* > 0.05) between groups. When normalized to FFM, there was a main effect age (*P* = 0.04), sex (*P* = 0.02), and an age × sex interaction (*P* < 0.01) for whole-body net balance at plateau (Fig. 4). F, AM, and AF (all, ~ 2.6 mg·kgFFM^− 1^·h^− 1^) had a greater (*P* < 0.05) net balance at plateau than M (~ 2.2 mg·kgFFM^− 1^·h^− 1^) with no further differences (*P* > 0.05) between groups.

## Discussion

The post-exercise consumption of dietary protein is important for recovery and growth (especially in youth) as it would help replenish exercise-induced amino acid oxidative losses [38] and provide the amino acid substrates to build new body proteins. It is well established in adults that carbohydrate and protein ingestion enhance muscle net balance after exercise [39]. For example, muscle protein breakdown is attenuated in response to exogenous amino acids [40] and/or the insulin response associated with moderate (i.e., ~ 30 g) carbohydrate ingestion [41, 42], whereas muscle protein synthesis is maximally stimulated by ~ 0.25 g protein·kg^− 1^ (or the equivalent of 0.06–0.08 g protein·kg^− 1^·h^− 1^) alone [6–8]. In contrast to muscle net balance, it has been suggested that there is no plateau in whole-body net balance in response to mixed meal ingestion [10–12]. This is suggested to be related to an insulin-induced suppression of whole-body protein breakdown combined with an enhanced retention of dietary amino acids within non-muscle tissue such as the splanchnic region [9]. This thesis is ostensibly supported by research demonstrating that muscle protein synthesis is not enhanced beyond the ingestion of ~ 0.25 g protein·kg^− 1^ in isolation [6, 7] or when combined within a mixed macronutrient meal [11], whereas whole-body net balance or leucine balance (i.e., leucine intake minus oxidation) increases linearly in adults up to ~ 0.93 g protein·kg^− 1^ [7, 11] and in children up to ~ 0.34 g protein·kg^− 1^ [17] when consumed as a single bolus. However, large bolus protein intakes are associated with an expansion of the plasma free amino acid pool, which ultimately is related to a saturation in the body’s ability to dispose of dietary amino acids through protein synthetic or oxidative pathways [6, 7, 11] and has been suggested to reflect an acute nutrient excess [43]. Metabolically, this sustained plasma amino acid concentration [44–46] would be broadly similar to constant oral feeding approaches [47, 48] such as that used in the present study. Using this logic, the bolus protein doses of 40 and 70 g used previously to support the lack of an anabolic plateau [11] would represent ~ 0.13 and 0.23 g·kg^− 1^·h^− 1^, respectively, and would fall within the intake ranges (0.02–0.25 g·kg^− 1^·h^− 1^) in the present study. With such a limited number of protein intakes, which in our hands would represent single intakes below and above the breakpoint, respectively, we argue that it is challenging to draw clear inferences as to the potential saturation of whole-body net balance. In contrast, the repeated design of the present IAAO study permitted us to investigate a greater dynamic range of protein intakes that resulted in a clear breakpoint in whole-body net balance in all groups studied. Given that our adult males plateaued after the ingestion of ~ 0.15 g·kg^− 1^·h^− 1^, it is possible that had others [11] included protein intakes above 70 g (i.e., ~ 0.93 g·kg^− 1^ bolus or 0.23 g·kg^− 1^·h^− 1^) a similar plateau in whole-body anabolism would have been apparent. Alternatively, bolus protein ingestion more robustly stimulates whole-body protein synthesis [49, 50], which could suggest our constant feeding approach may have slightly underestimated the full anabolic potential of constant mixed meal ingestion. While the differences in feeding pattern may have influenced the maximal relative protein intake at the breakpoint, we believe that the present data, as well as others that demonstrate plateaus in muscle [6–8], non-muscle [7], and whole-body protein synthesis [24] are supportive of a maximal anabolic response to mixed meal ingestion. Moreover, as the IAAO was developed to mimic a 12-h fed state similar to seminal 24-h intravenous infusion protocols [51], we believe our 5–8 h post-exercise period, which was dictated by model requirements but similar to previous rested oral infusion protocols [20], would provide a reasonable estimate of protein metabolism over both the immediate (i.e., < 4 h) and delayed (i.e., up to ~ 12 h) recovery time points with a constant feeding approach.

It has been suggested that the level at which protein intake becomes excessive is reflected by an increase in amino acid oxidation with graded protein intakes [52]. When dietary protein is consumed in excess of the rate at which it can be incorporated into body protein, excess amino acids are deaminated and oxidized [7], which is reflected by an increase in urea production for urinary nitrogen excretion [15]. Witard et al. [6] demonstrated an increase in urea production with 40 vs. 0, 10, and 20 g protein, which suggests that instead of incorporation into body protein, excess amino acids were metabolically partitioned toward oxidation and excretion [51]. Fromentin et al. [53] demonstrated that dietary amino acids (in the form of intrinsically labeled egg proteins) contributed modestly to increases in urinary end products, which suggests that an increase in urea excretion may reflect total body amino acid metabolism rather than the fate of dietary amino acids per se. Indeed, it has been suggested that the majority of dietary protein is used for the synthesis of new body proteins [49], suggesting amino acids arising from the turnover of old body proteins may represent the excess amino acid substrates that are diverted toward oxidation. Nevertheless, we observed robust breakpoints in whole-body net balance concomitant with a linear increase in urea/creatinine excretion, which we interpret collectively as being a plateau in whole-body anabolism.

During the pubertal growth spurt, a healthy normally developing child has a ~ 3-fold increase in growth velocity compared to the pre-pubertal period (< 10 y) [54], whereby the accrual of lean body mass can reach ~ 2.3 g·d^− 1^ (~ 96 mg·h^− 1^) in females and 3.8 g·d^− 1^ (~ 150 mg·h^− 1^) in males during this rapid growth period [55]. Moreover, youth who are physically active have been shown to have greater lean body mass when compared to their sedentary counterparts [19] and may be accruing lean mass at a greater rate. Importantly, adolescents involved in sports (e.g., ice hockey, basketball, soccer, etc.) have a greater BMI and lower total fat mass during adulthood [56], which suggests the high muscle forces and variable intensity nature of most team sports is important for lean mass (including bone) development across the lifespan. Provided that energy needs are met, this accrual of lean mass would ultimately be supported by dietary protein. We observed that whole-body net balance plateaued in active adolescents at a protein intake that was broadly similar between age groups and sexes. However, the mixed model y-intercept data demonstrated that adolescents had a greater whole-body net balance at very low protein intakes when compared to their adult counterparts, which is consistent with an enhanced anabolic sensitivity during adolescence [20]. Given that whole-body net balance is generally lower after exercise than muscle net balance [57], we speculate that maximizing whole-body net balance in adolescents would also be mirrored by similar changes in muscle net balance. Therefore, although moderate protein intake is sufficient to facilitate post-exercise anabolism in active adolescents, the rapid growth of this life stage translates into a greater anabolic sensitivity to suboptimal protein intakes than similarly active adults.

Unlike resistance exercise, variable intensity exercise is generally not associated with marked growth or muscle hypertrophy in weight-stable adults [3]. In contrast, the stop-and-go weight-bearing nature of the LIST and its ability to provide the recommended 60 min of daily moderate-to-vigorous physical activity would have provided a stimulus to enhance lean mass accrual in our adolescents [19]. After normalizing to the metabolically active FFM, both adolescent males and females exhibited an ~ 18% greater whole-body net balance when compared to adult males that would be consistent with an increased capacity for dietary protein assimilation, which could ultimately support the rapid growth during this critical developmental stage [55]. However, although physical activity can enhance the normal somatic gain of LBM during adolescence [19], it is unclear if the greater whole-body net balance at plateau and at low intakes in our adolescent population was due to their normal growth velocity, a greater anabolic effect of the LIST per se, or a combination of the two. Interestingly, adult females also had a greater whole-body net balance at plateau (normalized to FFM) when compared to adult males. In the present study, adult female participants were required to be in the luteal phase of their menstrual cycle, which is associated with uterus growth and a greater lysine requirement [58]. Thus, it is possible that the greater whole-body net balance at plateau in adult females compared to adult males was reflective of higher protein turnover during the luteal phase of their menstrual cycle. Therefore, while whole-body net balance demonstrated a clear plateau in active individuals regardless of age and sex, the capacity for dietary protein assimilation appears to be influenced by the metabolic state and growth potential of a population.

## Conclusions

We report that whole-body net balance plateaued in response to increasing protein intakes after a bout of variable intensity exercise, which is in agreement with our hypothesis and suggests that the anabolic response to post-exercise protein ingestion has a practical limit in both adults and adolescents. Importantly, we did not observe any statistical differences in the breakpoint between sexes within an age group, suggesting that postprandial protein recommendations to maximize whole-body net balance after exercise are primarily influenced by total body and fat-free mass. In further agreement with our hypothesis, the greater whole-body net balance at very low and optimal protein intakes in adolescents compared to adults highlight that active youth during the pubertal growth spurt have both a greater anabolic sensitivity and anabolic potential to mixed meal dietary protein ingestion during recovery from exercise.

## Abbreviations

AF: adolescent females
AM: adolescent males
F: adult females
FFM: fat-free mass
IAAO: indicator amino acid oxidation
LIST: Loughborough Intermittent Shuttle Test
M: adult males
